# Multimodal dataset using OCTA and fundus images for the study of diabetic retinopathy

**DOI:** 10.1016/j.dib.2024.110033

**Published:** 2024-01-11

**Authors:** Pooja Bidwai, Shilpa Gite, Aditi Gupta, Kishore Pahuja, Ketan Kotecha

**Affiliations:** a Symbiosis Centre for Applied Artificial Intelligence (SCAAI) Symbiosis Institute of Technology, Symbiosis International (Deemed University) (SIU), Lavale, Pune 412115, India; b Natasha Eye Care Shiv Sai Lane Pimple Saudagar, Pune, Maharashtra 411027, India; c Symbiosis Institute of Technology, Symbiosis International (Deemed University) (SIU), Lavale, Pune 412115, India

**Keywords:** Optical coherence tomography angiography, Fundus, Artificial intelligence, Non-proliferative Diabetic Retinopathy, A dataset combining OCTA and fundus images for the investigation of diabetic retinopathy

## Abstract

This article presents a Multimodal database consisting of 222 images of 76 people wherein 111 are OCTA images and 111 are color fundus images taken at the Natasha Eye Care and Research Institute of Pune Maharashtra, India. Nonmydriatic fundus images were acquired using a confocal SLO widefield fundus imaging Eidon machine. Nonmydriatic OCTA images were acquired using the Optovue Avanti Edition machine Initially, the clinical approach described in this article was used to obtain the retinal images. Following that, the dataset was categorized by two experienced eye specialists. To identify instances of Non-Proliferative Diabetic Retinopathy (NPDR) with their various stages, medical professionals and scholars can use this data. Research scholars and ophthalmologists can utilize the data created to develop the initial stages of automated identification techniques for diabetic retinopathy (DR).

Specifications Table

**Table:** 

Subject	Ophthalmology.
Specific subject area	Diabetic Retinopathy
Data format	Raw Images, Excel file
Type of data	1. Fundus images with dimensions 3680 × 3288, 96 dpi, and jpeg 2. OCTA (8 × 8 mm) images with dimensions 1596 × 990, 96 dpi and jpeg 3. Annotations of the classifications are provided in an EXCEL file (.xlsx).
Data collection	This dataset contains OCTA and Fundus images. Nonmydriatic fundus images were acquired using a confocal SLO widefield fundus imaging Eidon machine and Nonmydriatic OCTA images were acquired using the Optovue Avanti Edition machine. OCTA and Fundus images are classified into three categories NO DR signs, Mild DR, and Moderate DR.
Data source location	The dataset of 222 images was acquired at the Natasha Eye Care and Research Centre, Shiv Sai Lane, Pimple Saudagar Pune 411017.
Data accessibility	Repository name: Multimodal OCTA and Fundus Image dataset for detection of Diabetic Retinopathy (Version-1) Data identification number: https://doi.org/10.5281/zenodo.8375220 Direct URL to data: https://zenodo.org/record/8375220 Data is under a data usage agreement. Instructions for accessing these data: Access will be given on prior permission specifying the use of the dataset and with proper citation and DOI

## 1 Value of the Data

The multimodal dataset serves as a valuable resource for both the medical scientific community and informatics researchers. It plays a crucial role in several aspects:

• This multimodal data set is particularly helpful for training and conducting research by novice experts who are interested in the examination of DR at the early stage.
• As this is a multimodal dataset with Fundus and OCTA images, by comparing this with normal images, the data set can be used to study retinal anatomy and its physiological variations within diabetic and nondiabetic patients.
• Encouraging the creation of mathematical frameworks for automated medical diagnosis, particularly for the use of AI tools for detecting diabetic retinopathy (DR) early.
• Supporting the training of future physicians in identifying and analyzing various stages of DR using fundus and OCTA images [1,2].

## 2 Data Description

Retinal or fundus images play a crucial role in the identification of various eye-related disorders. These images capture essential structures within the eye, including blood vessels, the optic disc, and the macula. The information derived from these structures in retinal images is instrumental in diagnosing and treating a range of retinal conditions [3], [4], [5].

The Early Treatment Diabetic Retinopathy Study (ETDRS) classifies diabetic retinopathy into two main categories: Non-Proliferative Diabetic Retinopathy (NPDR) and Proliferative Diabetic Retinopathy (PDR). NPDR represents the early stage of the disease and further divides into mild, moderate, severe, and very severe. On the other hand, PDR is a more advanced form and is categorized into early and advanced stages. Medical experts have categorized the dataset into three distinct groups based on the pathological conditions associated with diabetic retinopathy. Consequently, the dataset is organized into three disease categories, including No DR signs, Mild (or early) NPDR, and Moderate NPDR For detailed information on the criteria used for classifying fundus images, please refer to [5]. The new dataset comprises 222 fundus and OCTA images of 76 persons where some patients have diabetes, and some patients don't have diabetes with corresponding labels and classification status available in the "Annotations of the classifications.xlsx" file.

Table 1 shows information available in the XLSX file with the following column description:

**Table 1:** Illustration of a sample representation of diabetic retinopathy (DR) labels contained within the XLSX file.

Person	Image number	Image format	Classification Status
1	L1.1	JPEG	NO DR
	L1.2	JPEG	NO DR
4	L4.1	JPEG	MILD DR
	L4.2	JPEG	MILD DR
	R4.3	JPEG	MILD DR
	R4.4	JPEG	MILD DR
6	R6.1	JPEG	MODERATE DR
	R6.2	JPEG	MODERATE DR
53	L53.1	JPEG	MODERATE DR
	L53.2	JPEG	MODERATE DR
	R53.3	JPEG	MILD DR
	R53.4	JPEG	MILD DR
72	L72.1	JPEG	MILD DR
	L72.2	JPEG	MILD DR
	R72.3	JPEG	MILD DR
	R72.4	JPEG	MILD DR

A. The “Person” attribute is the number of the patient in the dataset.

B. The “Image number” attribute gives the number of images for each patient.

**L** indicates Left Eye image and **R** indicates Right eye Image of the patient.

All odd number images e.g., LXX.1, RXX.3 are OCTA images and Even number Images e.g., LXX.2, RXX.4 are Fundus Images of Patients

C. The “Image Format” attribute shows the format of the image (.jpg).

D. The “Classification Status” attribute shows the classification of the DR.

Table 2 presents the database's categorization along with the number of OCTA and Fundus images allocated to each category.

**Table 2:** Categorization of the images within the database.

Classification	Number of images	OCTA	Fundus
NO DR	182	91	91
MILD DR	26	13	13
MODERATE DR	14	7	7

**Fig. 1:**
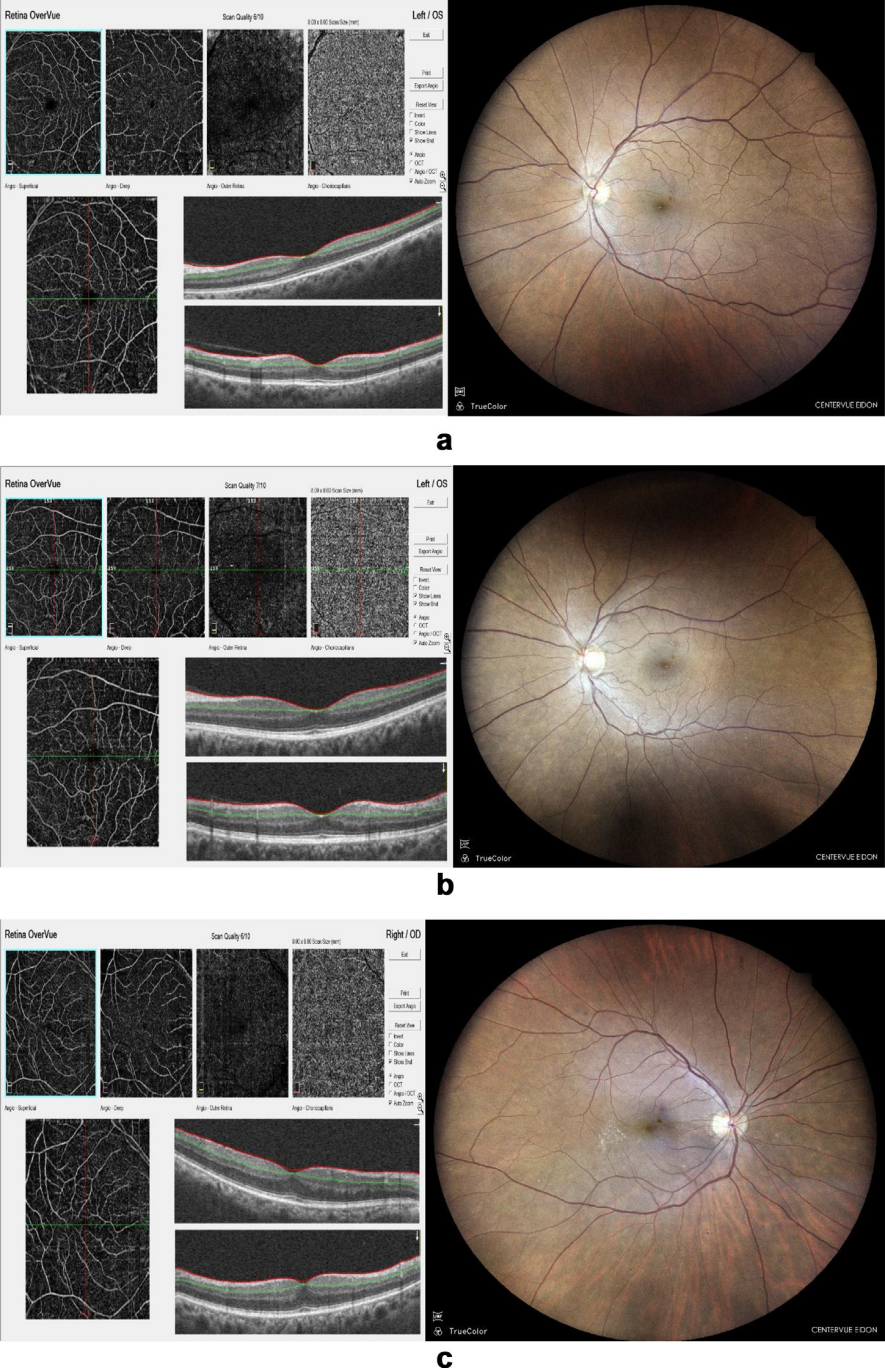
a OCTA and Fundus images with No signs of DR. Fig. 1b OCTA and Fundus images with Mild signs of DR. Fig. 1c OCTA and Fundus images with Moderate signs of DR.

Fig. 1 shows OCTA and Fundus images with different stages of DR.

Fig. 1a provides clear OCTA and Fundus images of the same person with no signs of DR. Microaneurysms, or any other lesions are absent in this case.

Fig. 1b provides OCTA and Fundus images of the same person with Mild signs of DR. Very few microaneurysms are visible in both the modalities in case of Mild DR.

Fig. 1c provides OCTA and Fundus images of the same person with Moderate signs of DR. In this type of DR, microaneurysms, along with retinal haemorrhages or exudates are visible.

## 3 Experimental Design, Materials and Methods

In this study total of 222 OCTA and Fundus images were obtained from individuals over the age of 30 years and above through a specific process. Cataract patients of similar age groups wherein some of them have diabetes and some of them don't have diabetes also some patients who have had diabetes for a very long time are considered. Post Operatively on Day-15 when individuals come for the second follow-up their OCTA and Fundus Scans are taken at Natasha Eye Care and Research Institute Pune. The described procedure is outlined as follows:

1. Patients undergo an interview, during which their information is documented in a patient file, including a checklist to document details regarding the patient's age and diabetes history. Additional health conditions or concurrent illnesses such as hypertension, ischemic heart disease (IHD), or the type of medication they are using, which may include oral pills, insulin, or a combination of both.
2. For advanced cases, systemic factors play a crucial role, including conditions like nephropathy, neuropathy, retinopathy, cardiomyopathy, vasculopathy, gastroparesis, and skin issues.
3. OCTA Images were obtained as 8 × 8 mm2 in size, centered on the macula, and images of superficial capillary plexus (SCP), Deep capillary plexus (DCP), and full thickness retinal slab are taken from Optovue Avanti Edition OCTA Machine with the Specifications**:** Machine Name: RTVue XR 100 Avanti Edition, FOV 32^o^ (H) x 22^o^ (V), Monochrome CCD Camera: WVGA 1/3^o^ CCD Format, NIR Illumination: 735 nm LED.
4. Fundus Scans are taken using Eidon machine with the Specifications as Centervue Eidon UWF Confocal SLO, Pupil size- 2.5mm, 110 ^o^ wide view scanning technique in automatic mode & 150^o^ in manual mode.
5. The determination of diabetic retinopathy stage is determined by a retina specialist. During the assessment of acquired retinographies, pictures that are out of focus, contain artifacts, or exhibit low image quality due to issues like low signal strength are excluded.
6. Additionally, retinographies from patients with any other retina treatment, patients with macular edema [6], and macular drusens, Proliferative Diabetic Retinopathy Patients, post-laser patients, and PVD (Posterior Vitreous Detachments) patients are not incorporated.

## Limitations

Describing the research and obtaining patients' consent poses a significant challenge [[7], [8]]. Additionally, performing an OCTA scan requires expert guidance because it necessitates more patient cooperation to achieve optimal clarity. This study is limited to 222 multimodal images, which includes 111 OCTA images and 111 Fundus images of 76 patients.

## Ethics Statement

The study was approved by the Symbiosis Institutional Ethics Committee (BHR) SIU with approval number SIU/IEC/583. Every patient provided informed consent for both treatment and examination taken by the Department of Ophthalmology at Natasha Eye Care and Research Institute. Data is protected by a data usage agreement. The patients' identities remain undisclosed, and the confidentiality of their medical conditions is rigorously maintained.

## CRediT authorship contribution statement

**Pooja Bidwai:** Conceptualization, Investigation, Data curation, Writing – original draft, Visualization. **Shilpa Gite:** Conceptualization, Investigation, Visualization, Writing – review & editing. **Aditi Gupta:** Investigation, Resources, Data curation, Writing – review & editing, Visualization. **Kishore Pahuja:** Conceptualization, Validation, Resources, Project administration. **Ketan Kotecha:** Validation, Writing – review & editing.

## Data Availability

Multimodal OCTA and Fundus Image dataset for detection of Diabetic Retinopathy (Version-1) (Original data) (Multimodal OCTA and Fundus Image dataset for detection of Diabetic Retinopathy (Version-1))
